# Supplementary figures and images for: The inconsistent microbiota of Budu, the Malaysian fermented anchovy sauce, revealed through 16S amplicon sequencing
Source: PeerJ. 2021 Oct 28;9:e12345. doi: 10.7717/peerj.12345 (PMC8557686; doi:10.7717/peerj.12345)

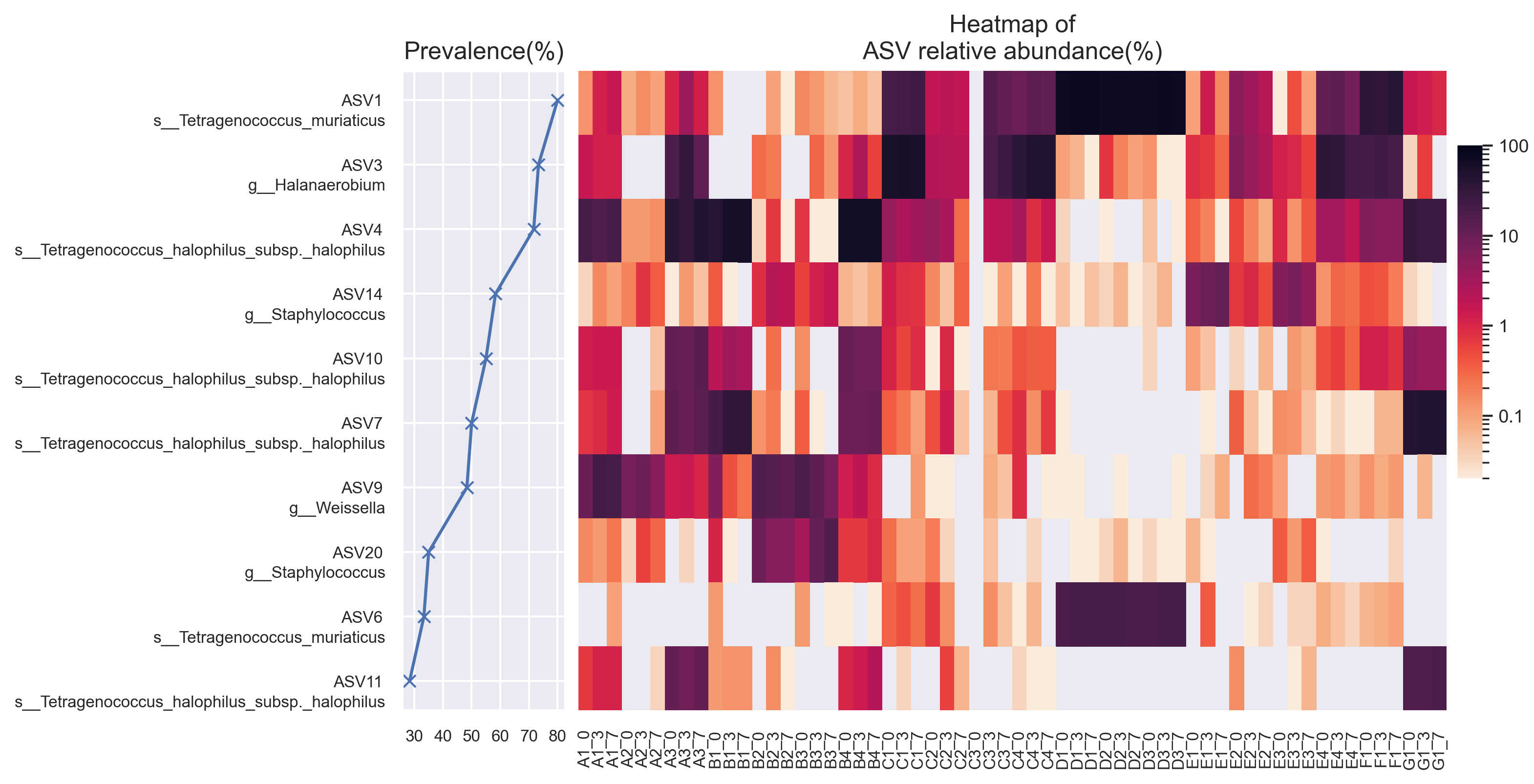

Supplement: Supplemental Information 1 [file peerj-09-12345-s001.png]

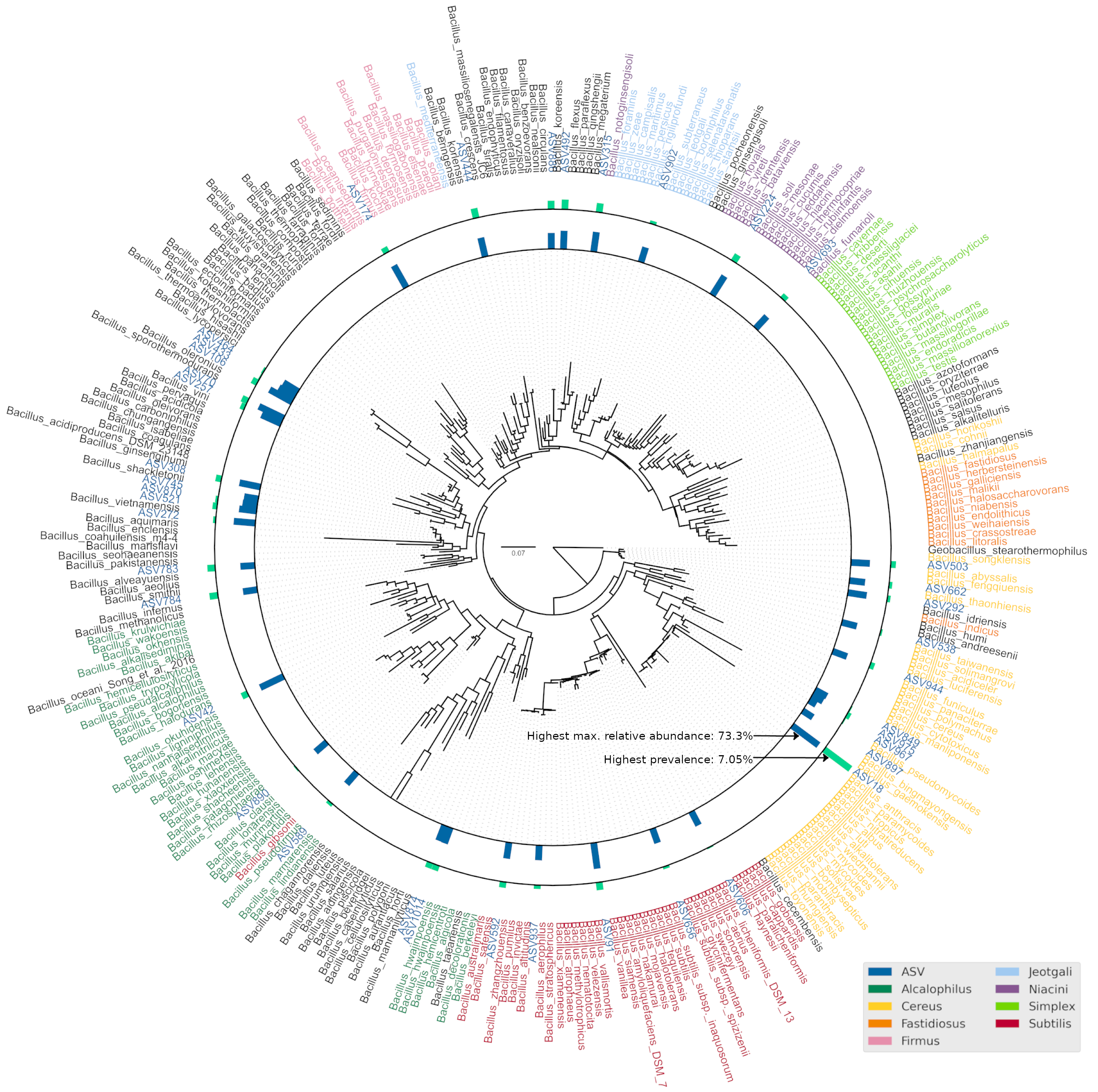

Supplement: Supplemental Information 2 — Bar plots represent prevalence (blue, max. = 73.33%) and maximum relative abundance of ASV (green, max. = 7.05%). ASV18 was the most abundant and prevalent Bacillus ASV. Leaf nodes are colored according to Patel and Gupta (Patel & Gupta, 2020) Bacillus clade designation. The phylogenetic tree was rooted to Geobacillus stereothermophilus as outgroup. [file peerj-09-12345-s002.png]

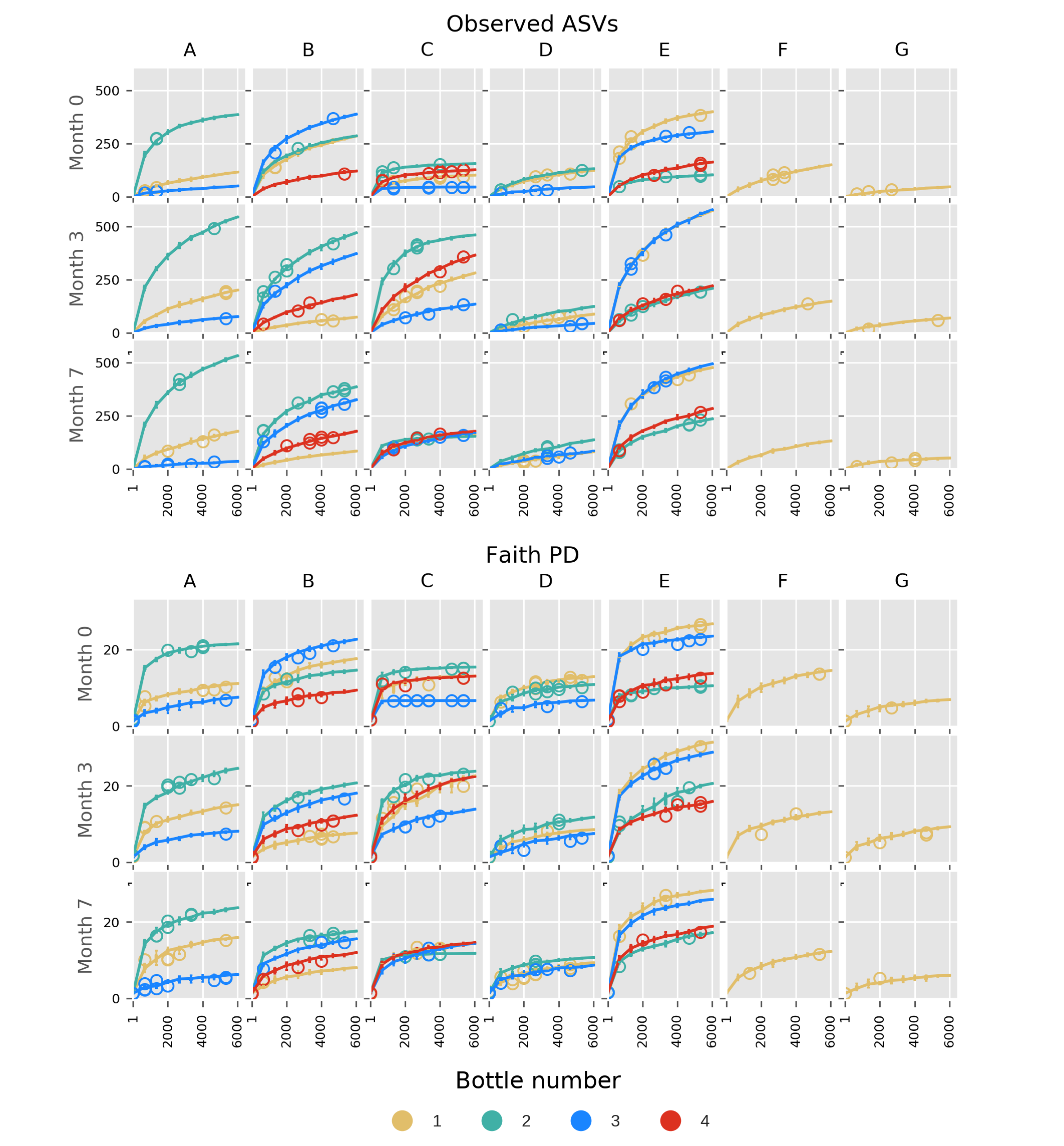

Supplement: Supplemental Information 3 — The interquartile ranges are shown. Circles indicate outlier alpha diversity calculations. [file peerj-09-12345-s003.png]

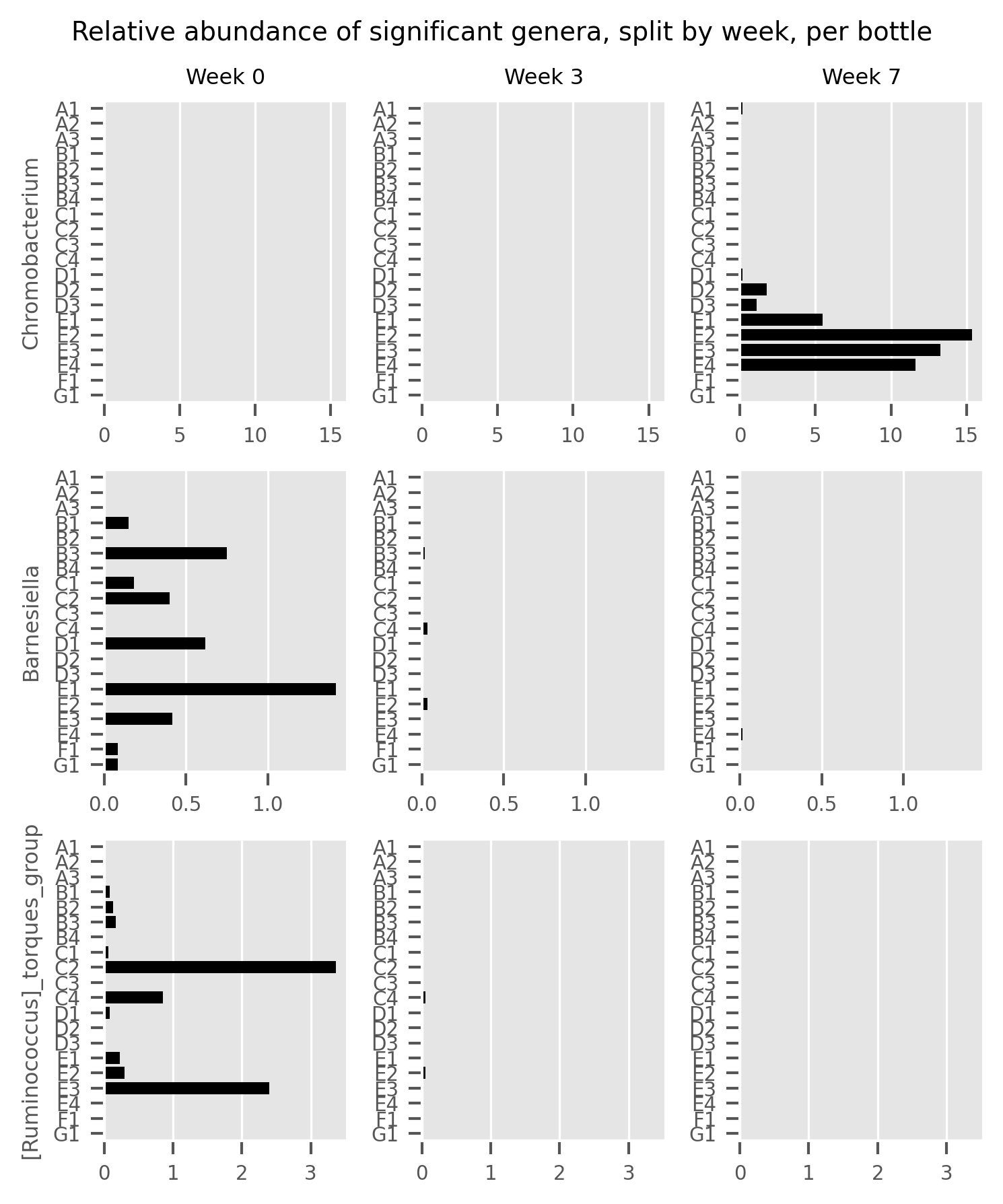

Supplement: Supplemental Information 4 [file peerj-09-12345-s004.png]
